# Fibroblast Growth Factor 10 in Pancreas Development and Pancreatic Cancer

**DOI:** 10.3389/fgene.2018.00482

**Published:** 2018-10-29

**Authors:** Rodrick Ndlovu, Lian-Cheng Deng, Jin Wu, Xiao-Kun Li, Jin-San Zhang

**Affiliations:** ^1^College of Life and Environmental Sciences, Wenzhou University, Wenzhou, China; ^2^School of Pharmaceutical Sciences, Wenzhou Medical University, Wenzhou, China; ^3^Centre for Precision Medicine, the First Affiliated Hospital, Wenzhou Medical University, Wenzhou, China

**Keywords:** FGF10, FGFR2b, SOX9, pancreas development, pancreatic adenocarcinoma, mesenchyme, epithelium

## Abstract

The tenacious prevalence of human pancreatic diseases such as diabetes mellitus and adenocarcinoma has prompted huge research interest in better understanding of pancreatic organogenesis. The plethora of signaling pathways involved in pancreas development is activated in a highly coordinated manner to assure unmitigated development and morphogenesis in vertebrates. Therefore, a complex mesenchymal–epithelial signaling network has been implicated to play a pivotal role in organogenesis through its interactions with other germ layers, specifically the endoderm. The Fibroblast Growth Factor Receptor FGFR2-IIIb splicing isoform (FGFR2b) and its high affinity ligand Fibroblast Growth Factor 10 (FGF10) are expressed in the epithelium and mesenchyme, respectively, and therefore are well positioned to transmit mesenchymal to epithelial signaling. FGF10 is a typical paracrine FGF and chiefly mediates biological responses by activating FGFR2b with heparin/heparan sulfate (HS) as cofactor. A substantial number of studies using genetically engineered mouse models have demonstrated an essential role of FGF10 in the development of many organs and tissues including the pancreas. During mouse embryonic development, FGF10 signaling is crucial for epithelial cell proliferation, maintenance of progenitor cell fate and branching morphogenesis in the pancreas. FGF10 is also implicated in pancreatic cancer, and that overexpression of FGFR2b is associated with metastatic invasion. A thorough understanding of FGF10 signaling machinery and its crosstalk with other pathways in development and pathological states may provide novel opportunities for pancreatic cancer targeted therapy and regenerative medicine.

## Introduction

The Fibroblast Growth Factor (FGF) family of peptides and the corresponding family of receptor tyrosine kinases (RTKs) collectively constitute one of the most adaptable, complex, and diverse growth factor signaling systems that are involved in many developmental and repair processes in virtually all vertebrate and invertebrate tissues and cells (Goetz and Mohammadi, 2013). Currently, the mammalian FGF nomenclature encompasses FGF1 to FGF23, comprising of secreted signaling proteins that transduce signals via their specific FGF receptors (FGFRs), and intracellular FGFs that serve as cofactors for voltage-gated sodium channels. These ligands are divided and grouped into seven subfamilies based on phylogenetic analysis, sequence similarities, and function (Goetz et al., 2009; Ornitz and Itoh, 2015).

FGFR family of RTKs comprises of FGFR1, FGFR2, FGFR3, and FGFR4. As the name suggests, FGFRs bind to members of secreted FGFs along with the sequential formation of complexes with heparin/heparan sulfate (HS) cofactor-proteoglycans to propagate downstream signal transduction pathways, which include activation of PLCγ, MAPK, AKT, and STAT cascades. At the cellular level, paracrine FGF-FGFR-HS signaling engages in vital roles in regulating cell proliferation, migration, survival, and differentiation during the development of the embryo (Kato and Sekine, 1999; Ornitz and Itoh, 2015).

FGF10, a FGF7 subfamily member, is a typical paracrine FGF and chiefly mediates its biological responses by activating FGFR2b. FGF10 is a potent morphogen and plays a crucial role in transmitting mesenchyme signaling to the epithelium. Genetic ablation of FGF10 in mice results in gross developmental defects characterized by agenesis and dysgenesis in a variety of organs and tissues highlighting an essential role of FGF10 signaling for the development of multiple organs including the pancreas (Bellusci et al., 1997; Bhushan et al., 2001; Itoh and Ohta, 2014). Although not as widely explored as in the development field, there is strong evidence suggesting that FGF10 is also involved in the pancreatic carcinogenesis (Nomura et al., 2008). Herein, we summarize the recent information about the involvement of FGF10 in pancreas development and diseases with a focus on pancreatic cancer.

## Fgf10 Signaling Machinery

Alternative splicing of the extracellular IgIII loop of FGFR1-3 generates IIIb- and IIIc-variants of the receptors. Tissue- and cell-specific expression of these isoforms and modification in binding properties for the FGF ligands confer signaling specificity and functional diversity in regulating interactions in embryonic development, tissue homeostasis, repair, and cancer (Itoh and Ohta, 2014). FGFR2 generates two isoforms via alternative splicing, FGFR2b, predominantly expressed in epithelial cells and FGFR2c, chiefly expressed in mesenchymal cells. A distinct feature of the FGF7 subfamily is the preferential binding to their cognate receptor FGFR2b in a HS dependent manner in contrast to most other FGFs predominantly interacting with FGFR2c (Givol and Yayon, 1992; Orr-Urtreger et al., 1993; Lindahl et al., 1998; Holzmann et al., 2012).

**FIGURE 1:**
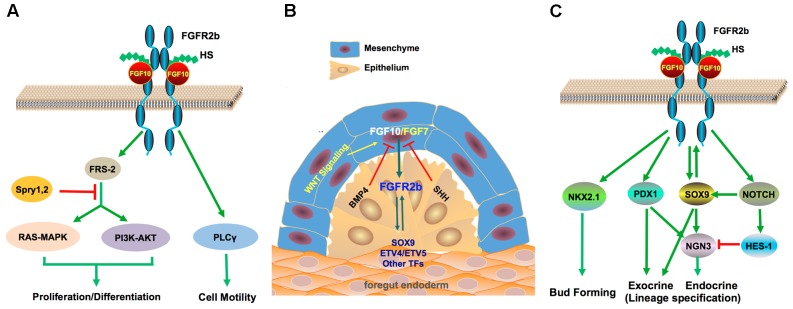
FGF10 signaling and its key crosstalk during pancreas development. **(A)** FGF10 is a high affinity ligand for FGFR2b. FGF10 interacts with FGFR2b with HS as cofactor and induces activation of the RAS-MAPK, PI3K-AKT, and PLCγ pathways, which mediate cell differentiation, proliferation, and motility. SPRYs are negative regulators of the RAS-MAPK and PI3K-AKT pathways. **(B)** FGF10 mediates mesenchyme to epithelial signaling through crosstalk with several key developmental pathways including WNT factors, BMP and SHH, which are important in pancreatic cell fate specification and branching morphogenesis. BMP signaling is required for the normal development of the mesenchyme as well as the epithelium. **(C)** FGF10 has a crucial role in epithelial branching morphogenesis through crosstalk with several key TFs and regulators for pancreas development. The FGF10/FGFR2b/SOX9 regulatory loop promotes proliferation and maintains pancreatic fate in pancreatic progenitors.

Formation of the FGF10-FGFR2b-HS (2:2:2) ternary complex results in the phosphorylation of intracellular tyrosine residues in FGFRs (Figure 1A). Phosphorylated FGFRs activate FGFR substrate 2α(FRS2α) and phospholipase Cγ (PLCγ1), which mediate cell motility (Zhang et al., 2006; Itoh and Ohta, 2014). FRS2α, in turn, facilitates the activation of RAS-MAPK or PI3K-AKT and PLCγ activates protein kinase C. The RAS-MAPK and PI3K-AKT pathways are predominantly involved in mitogenic cell responses or cell survival and are subjected to negative regulation by SPRY1 and SPRY2 (Tefft et al., 2002; Zhang et al., 2006). These signaling cascades mediate a diverse range of biological outcomes that define FGF10/FGFR2b dependent signaling (Figure 1A). The spatiotemporal expression and activity of FGFs and FGFR isoforms is additionally enhanced by the diversity of HS structures, which are also involved in developmental processes, insinuating that tissue-specific HS regulates FGF signaling (Lindahl et al., 1998; Makarenkova et al., 2009).

Interestingly, although FGF7 and FGF10 share a common receptor, expression in mesenchyme and the ability to promote proliferation of embryonic pancreatic epithelial cells *in vitro* (Ye et al., 2005), the phenotypes of their knockout mice are drastically different in that FGF7 null mice are born with no obvious abnormalities (Guo et al., 1996), whereas FGF10 knockout mice die at birth with major defects in multiple organs such as lung agenesis and pancreas dysgenesis (Min et al., 1998; Sekine et al., 1999; Ohuchi et al., 2000; Itoh and Ornitz, 2011). Based on a sophisticated quantitative proteomics approach, Francavilla et al. (2013) uncovered a fascinating ligand-dependent mechanism for the control of FGFR2b turnover and signaling outputs. FGF7 stimulation leads to FGFR2b degradation and, ultimately, cell proliferation, whereas FGF10 triggers additional phosphorylation on Y734 of FGFR2b leading to its recruitment of PI3K and SH3BP4 to promote receptor recycling and sustained signaling.

Zinkle and Mohammadi recently proposed a threshold model for RTK signaling specificity and cell fate determination (Makarenkova et al., 2009; Francavilla et al., 2013; Zinkle and Mohammadi, 2018). It is suggested that the intensity and duration of signaling via FGFR2b is dependent on the phosphorylation of Y734 within the kinase domain. Higher affinity of FGF10 for binding both FGFR2b and the co-receptor HS (Makarenkova et al., 2009) generates a more robust interaction than FGF7-FGFR2b dimers, therefore propagates more sustained MAPK signal that leads to cell proliferation and migration whilst FGF7 propagates a transient MAPK signal that leads to cell proliferation. It is conceivable that the difference in ligand-induced dimer stability distinguishes FGF7 from FGF10 on the choice and durability of intracellular pathways, which may well contribute to their functional discrepancies on branching morphogenesis during embryonic development.

## Fgf10 in Pancreas Development

The pancreas is an endoderm-derived glandular organ that partakes in the regulation of glucose homeostasis and nutrient uptake through the concerted functions of its endocrine and exocrine compartments, respectively (Edlund, 1999; Shih et al., 2013). Early mouse pancreas development has two characteristic periods: a primary transition (E9.5–12.5) that is characterized by rapid cell proliferation and histogenesis and a secondary transition (E12,5-birth) after rotation of the gut at E12.5 that is chiefly characterized by cytodifferentiation and formation of the significant intracellular organelles of the adult pancreatic cell (Pictet et al., 1972; Jorgensen et al., 2007; Benitez et al., 2012).

The mesenchyme is critical for the growth of all pancreatic lineages (Landsman et al., 2011). Reports indicate that FGF signaling derived from the surrounding mesenchymal tissue is pivotal for the genesis of specific cellular domains (Hart et al., 2003; Zhou et al., 2007). FGF10, as a mesenchymal factor, has an indispensable role in ensuring the development of the pancreatic epithelium, which gives rise to the functional endocrine and exocrine cell types (Bhushan et al., 2001; Elghazi et al., 2002; Hart et al., 2003; Norgaard et al., 2003). To ascertain the role of FGF10 in pancreas development, Bhushan et al. (2001) demonstrated that FGF10 expressed from E9.5 until E11.5 in mice is vital for pancreas growth and differentiation of Pdx1 ^+^ epithelial precursor cells. The absence of this mesenchymal protein led to pancreatic hypoplasia (Bhushan et al., 2001). Furthermore, the pancreata of *Fgfr2b*^-/-^ mutant mice were smaller than the wild type littermates with pancreatic duct cell proliferation notably reduced (Miralles et al., 1999; Pulkkinen et al., 2003). FGF10 signaling predominantly targets the adjacent tissue due to its paracrine nature, hence in *Fgf10* null mutant mice, the pancreatic progenitor cells are diminished even before the onset of secondary transition. The few exocrine cells present do undergo differentiation and form acinar structures (Bhushan et al., 2001). Mice deficient in FGFR2b exhibit mild phenotypes comparable to the FGF10 null mice with differentiation of both pancreas compartments and consequent reduction of organ size (Miralles et al., 1999; Pulkkinen et al., 2003).

While many literature sources substantiate the role of FGF10 in epithelial development, the expression levels of the protein decrease to almost unperceivable levels at E13.5 in mice (Bhushan et al., 2001; Elghazi et al., 2002; Kobberup et al., 2010). Explant studies in mice involving pharmacological inhibition of FGF signaling proved that FGF10 is dispensable at later stages of gestation, implying that different epithelial cell types not only depend on FGF10 signals but also on other (same or distinct) mesenchymal factors (Greggio et al., 2013). Possibly, FGF10′s primary role is vital for the initial stage of progenitor growth, then might work in concert with other mesenchymal derived factors or signaling pathways.

## Fgf10 Crosstalk With Other Signaling Pathways

The mesenchyme is a source of cell-extrinsic signals that promotes pancreatic specification, yet limits differentiation, so as to allow expansion of the pancreatic epithelium. Besides FGFs, other mesenchymal signals that promote growth of the pancreatic epithelium include WNT factors (Jonckheere et al., 2008), Retinoic Acid (RA) (Stafford et al., 2006), BMP (Ahnfelt-Ronne et al., 2010), and the TGF-β pathway (Crisera et al., 2000; Figure 1B).

FGFs and WNT factors are known to act in synergy to promote proliferation in a variety of developmental systems (ten Berge et al., 2008; Afelik et al., 2015). Canonical WNT signaling is a mediator of epithelial to mesenchymal signaling, several WNT ligands plus frizzled (FRZ) receptors (e.g., WNT2b, WNT7b, and FRZ2-9) are expressed by both the mesenchyme and pancreatic epithelial cells during organogenesis (Heller et al., 2002; Afelik et al., 2015). Comparable phenotypes are observed between *Pdx1/Frz8CRD* (dominant-negative frizzled 8 receptor) and *Pdx1/Fgf10* null neonates revealing pancreatic hypoplasia, as early as E14, further implying a role for both signaling pathways in pancreatic growth (Papadopoulou and Edlund, 2005; Jonckheere et al., 2008).

RA signaling is also an indispensable mediator of mesenchymal function. In the lung, mesenchyme RA signaling has been implicated in the induction of FGF10 (Desai et al., 2004). Furthermore, absence of RA signaling leads to pancreatic hypoplasia (severe in the dorsal pancreas) (Martin et al., 2005). In an effort to produce functional β cells from endoderm derived human embryonic stem (hES) cells, Mfopou et al. (2010) exposed these hES cells to noggin and RA, followed by FGF10 during early stage of induction, and successfully generated pancreatic cells, the majority of them are *Pdx1* ^+^ that coexpressed FOXA2, HNF6, and SOX9.

Unmitigated differentiation of the mesenchyme, which further ensures proper epithelial development, is reliant on many signaling molecules except members of the Hedgehog family from the early pancreatic niche (Kawahira et al., 2005). Ectopic expression of Sonic Hedgehog (SHH) in mice driven by the *Pdx1* promoter results in differentiation of the pancreatic mesenchyme into smooth muscle and the epithelium assumes an intestinal fate with the generation of few early endocrine cell types (Apelqvist et al., 1997). SHH is also implicated in repressing expression of *Fgf10* (Figure 1B; Bhushan et al., 2001).

## Transcription Factors Implicated in Fgf10 Signaling

Genetic lineage tracing experiments have elucidated that cell clusters committed to adopting the pancreatic lineage express the transcription factor (TF) PDX1 (Pancreatic and duodenal homeobox 1) and PTF1a (Pancreas transcription factor 1). Ablation of either *Pdx1* or *Ptf1a* causes pancreatic agenesis or diabetes and wide gastro-duodenal deformations (Offield et al., 1996; Stoffers et al., 1997; Kawaguchi et al., 2002; Burlison et al., 2008; Fukuda et al., 2008).

After the establishment of the pancreatic anlage, a gene regulatory network is established with *Pdx1* at the focal apex in order to maintain pancreatic identity (Shih et al., 2015). PDX1 exhibits an extensive cross-regulation network between individual TFs and FGFs such as FGF10; however, sustentation of the pancreatic lineage requires high levels of PDX1 (Shih et al., 2015). Augmentation of PDX1 expression levels is supplemented by PTF1a, which binds to enhancer elements of PDX1 (Wiebe et al., 2007), whilst FGF10 is required to maintain the PDX1 ^+^ expressing progenitor cell pool (Figure 1C; Bhushan et al., 2001).

Genetic lineage tracing has shown that multipotent progenitor cells (MPCs) can be similarly defined by several TFs such as SOX9, HNF6, NKX2.2, HNF1β, HES1, CAP1, and NKX6.1. At this juncture, MPCs not only have the potential to self-renew, but also can differentiate to form exocrine and endocrine progenitors with PDX1 functioning as the central node (Zhou et al., 2007; Pan and Wright, 2011; Seymour, 2014).

The SOX9 interacts with the FGF signaling pathway in concert with PDX1 to maintain both expansion (in a dosage-dependent manner) and organ identity of MPCs (Shih et al., 2013). SOX9 and PDX1 co-regulate the pancreatic versus intestinal lineage choice, ablation of both genes causes MPCs to embrace an alternative hepatic fate (Seymour et al., 2012; Shih et al., 2015). In mice, SOX9, FGFR2b, and FGF10 form a feed-forward expression loop; SOX9 cell-autonomously maintains FGFR2b expression, which in turn, augments its epithelial receptivity to FGF10, whilst FGF10 maintains SOX9 expression (Figure 1C). Hence nullification of any component in this loop leads to pancreatic hypoplasia and loss of both SOX9 plus FGFR2b in FGF10-deficient MPCs leads to hepatic reprogramming (Seymour et al., 2012).

## Fgf10 Mediates Pancreatic Cell Fate

Spatial and temporal regulation of gene function is vital in the modeling of specialized cell types from a field of competent cells. FGF10 is known to maintain progenitor cells in an undifferentiated state to allow subsequent proliferation, ectopic expression results in a hyperplastic pancreas. Nascent emergent patterns of budding cells are additionally controlled by conserved developmental pathways such as the NOTCH signaling via lateral inhibition/specification in order to integrate terminal differentiation in FGF10 signaling. FGF10-positive progenitor cells express NOTCH1 and NOTCH2, the NOTCH-ligand genes JAG1 and JAG*2*, as well as the NOTCH target gene HES1 (Murtaugh et al., 2003; Norgaard et al., 2003; Miralles et al., 2006).

During the primary transition, NOTCH and FGF10 signaling are predominantly involved in restricting premature endocrine differentiation and maintenance of the progenitor state. Ablation of Notch target genes such as *Dll1* (Hrabe de Angelis et al., 1997), *Rbp-jk* (Fujikura et al., 2006), or *Hes1* (Jensen et al., 2000) results in an increase of NGN3 ^+^ cells, leading to premature differentiation of the MPCs into glucagon ^+^-cells (Apelqvist et al., 1999) and p57-expressing progenitor cells, which undergo premature cell cycle exit evident with the expression of a hypoplastic pancreas (Georgia et al., 2006). This phenotype is comparable to *Fgf10* and *Sox9* null mutant mice. HES1 is known to repress both the transcriptional activation of *Ngn3* and the cyclin kinase inhibitor *P57* (Figure 1C; Georgia et al., 2006).

SOX9 is a positive regulator of NGN3 in a dosage-dependent manner, and is expressed chiefly in trunk progenitor cells and its depletion results in the reduction of NGN3 ^+^ cells. This suggests that there may exist a complicated but well-organized regulatory system involving FGF10, FGFR2b, NOTCH, HES1, SOX9, and NGN3 that controls endocrine differentiation and maintenance of progenitor cells (Miralles et al., 2006; Kobberup et al., 2010; Gouzi et al., 2011; Afelik and Jensen, 2013; Shih et al., 2015). It can be postulated that both FGF10 and NOTCH signaling pathways are critical for the establishment of two cell lineages:

(i) NGN3 ^+^ cells that form the early α-cells.
(ii) NGN3 ^+^ that will remain proliferative and available to differentiate to other endocrine cell types (Apelqvist et al., 1999; Jensen et al., 2000; Miralles et al., 2006; Kobberup et al., 2010; Afelik and Jensen, 2013).

Ectopic expression of *Fgf10* from E10.5 to E13.5 leads to nearly complete loss of endocrine and ductal differentiation (Kobberup et al., 2010). This, in turn, favors the exocrine lineage because of the lack of competence to form the endocrine cell lineage. Furthermore, exocrine (acinar) differentiation has been observed to occur in FGF10 null mutant mice implying that FGF10 does not entirely control exocrine differentiation but rather it is permissive toward exocrine lineage fate (Miralles et al., 1999; Bhushan et al., 2001; Kobberup et al., 2010). This is observed with sustained expression of PTF1A in both *Fgf10*^-/-^ mutant and wild type mice though reports have indicated that downstream effectors of FGF10, such as *Etv4* and *Etv5*, influence expression of PTF1A (Figure 1C; Dong et al., 2007; Kobberup et al., 2007, 2010).

Cellular proliferation and differentiation are mutually exclusive events; hence overexpression of FGF10 beyond the primary transition perturbs differentiation of endocrine and ductal cell types. At this stage, progenitor cells typically co-express PDX1, NKX6.1, and PTF1A, failure of endocrine cell formation leads to diabetes in mice (Hart et al., 2003; Petri et al., 2006; Kobberup et al., 2010). FGF10 signaling via FGFR2b is at the expense of endocrine cellular differentiation (Celli et al., 1998; Miralles et al., 1999; Pulkkinen et al., 2003). By understanding the exact timing of the competence window toward endocrine fate, FGF10 could be best exploited in cell-based therapeutic strategies to combat diabetes (Madsen and Serup, 2006).

## Fgf10 -Fgfr2B in Pancreatic Ductal Adenocarcinoma

**FIGURE 2:**
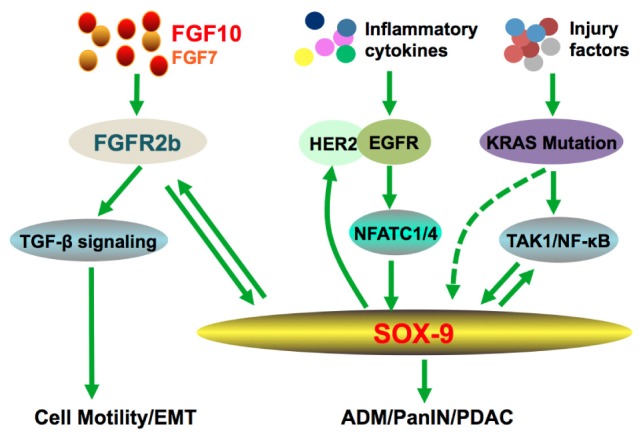
Crosstalk of FGF10 during pancreatic cancer. Interactions of FGF10 with TGF-β pathway promote EMT and cancer cell invasion. The positive feedback loops between FGF10-SOX9, KRAS/NF-κB-SOX9, and ERBB-SOX9, respectively, are enhanced under inflammatory condition, which contributes to PDAC initiation and progression.

Pancreatic ductal adenocarcinoma (PDAC) is the most common exocrine malignancy and represents one of the deadliest diseases with high mortality due to difficulties in its early diagnosis, metastasis and intrinsic resistance to conventional chemoradiotherapy. At a molecular level, cancer cells in PDAC are often characterized by mutations in the KRAS oncogene, SMAD4, and TP53. Several FGFs and FGFRs are expressed in stromal cells scattered around pancreatic cancer cells and their expression levels have been linked to increased cancer motility, proliferation and metastatic invasion (Kalluri and Zeisberg, 2006; Ying et al., 2016). FGF7 and 10 are both expressed in stromal cells surrounding cancer cells. Regardless of the high homology the latter induces cell migration and invasion whilst the former stimulates cell proliferation. FGF10-FGFR2b signaling induces the expression of type1-matrix metalloproteinase and *TGF-β1* genes (Nomura et al., 2008), these genes are related to cell motility (Friess et al., 1993; Seiki, 2003). Moreover, FGF10-FGFR2b signaling induced the secretion of TGF-β1, a crucial regulator of epithelial to mesenchymal transition (Figure 2; Moustakas and Heldin, 2007; Nomura et al., 2008).

A hallmark genetic alteration of PDAC is the high frequency mutation of KRAS. Numerous studies demonstrate that oncogenic KRAS mutations induce Acinar-to-ductal metaplasia (ADM), pancreatic intraepithelial neoplasia (PanIN), and eventually PDAC. Significantly, SOX9 is imperative for KRAS^G12D^-mediated ADM and PanIN formation (Kopp et al., 2012). A more recent study demonstrated that KRAS can independently induce SOX9 expression and promoted its nuclear translocation and transcriptional activity, which plays a positive role in the proliferation of PDAC cells (Zhou et al., 2018).

Our recent studies further showed that SOX9 could be induced by NFATC1 and NFATC4 in response to EGFR activation and pancreatitis, which promote ADM and PanIN (Chen et al., 2015; Hessmann et al., 2016). In a separate study, SOX9 is reported to stimulate expression of several members of the ERBB pathway, and is required for ERBB signaling activity to promote pancreatic tumorigenesis (Grimont et al., 2015). These studies further consolidate SOX9 as a central player in pancreatic adenocarcinoma via promoting ADM, particularly in the context of oncogenic KRAS and pancreatitis to accelerate development of premalignant lesions and PDAC (Figure 2). Therefore, three positive feedback loops have emerged from these studies (Figure 2): (1) FGF10/FGFR2/SOX9 inter-dependent expression is also present in a subset of PDAC patients (Seymour et al., 2012; O’Sullivan et al., 2017); (2) EGFR, via activation of NFATC1 and NFATC4, promotes SOX9 expression, whereas activated SOX9 stimulates ERBB2 protein expression (Chen et al., 2015; Grimont et al., 2015; Hessmann et al., 2016); (3) Oncogenic KRAS via TAK1/NF-κβ promotes SOX9 expression/activation, and SOX9 in turn enhances NF-κβ activity (Zhou et al., 2018). These findings open new perspectives for precision therapeutic strategies targeting specific cancer-driven signaling molecules such as ERBB2 or FGFR2.

## Conclusion and Perspective

Animal models lacking each of the secreted FGFs have been developed with diverse phenotypes ranging from mild abnormality in adult physiology to early embryonic lethality. Only three FGFs (FGF9, FGF10, and FGF18) upon knockout result in early postnatal lethality due to severe developmental defects in multiple organs. While *Fgf9* and *Fgf18* are essential for the development of mesenchymal components, numerous studies highlight FGF10 as an indispensable mesenchyme to epithelium signal required for the development of epithelial components in multiple organs. Despite the interesting observations from previous reports, research on FGF10/FGFR2b in the pancreas is lagging behind compared to some other organs such as the lung. There remain some critical questions unanswered regarding how FGF/FGFR2b signaling influence acinar and ductal specification (e.g., further proliferation and differentiation from the progenitor cells), as well as its impact on the endocrine system remain largely unexplored. More elegant and specifically targeted genetic models allowing better spatiotemporal manipulation of gene expression will be essential to better address these questions. During both embryonic development and oncogenic process, FGF10 acquires the ability for unique crosstalk with other pathways as exemplified by its inter-dependent expression with SOX9, which may represent a key knot linking oncogenic KRAS, inflammation and other growth factor signaling. Understanding of FGF10 signaling machinery and its crosstalk with other pathways may provide novel opportunities for PDAC precision therapy and regenerative medicine.

## Author Contributions

RN, L-CD, JW, X-KL, and J-SZ conceived the study. RN and J-SZ wrote the manuscript. RN, L-CD, JW and J-SZ designed and drew the figures. J-SZ designed and edited the manuscript. J-SZ and X-KL supervised the study and acquired funding.

## Conflict of Interest Statement

The authors declare that the research was conducted in the absence of any commercial or financial relationships that could be construed as a potential conflict of interest.

## References

[B1] Afelik S. Jensen J. (2013). Notch signaling in the pancreas: patterning and cell fate specification. *Wiley Interdiscip. Rev. Dev. Biol.* 2 531–544. 10.1002/wdev.99 24014421

[B2] Afelik S. Pool B. Schmerr M. Penton C. Jensen J. (2015). Wnt7b is required for epithelial progenitor growth and operates during epithelial-to-mesenchymal signaling in pancreatic development. *Dev. Biol.* 399 204–217. 10.1016/j.ydbio.2014.12.031 25576928

[B3] Ahnfelt-Ronne J. Ravassard P. Pardanaud-Glavieux C. Scharfmann R. Serup P. (2010). Mesenchymal bone morphogenetic protein signaling is required for normal pancreas development. *Diabetes* 59 1948–1956. 10.2337/db09-1010 20522595 PMC2911072

[B4] Apelqvist A. Ahlgren U. Edlund H. (1997). Sonic hedgehog directs specialised mesoderm differentiation in the intestine and pancreas. *Curr. Biol.* 7 801–804. 10.1016/S0960-9822(06)00340-X 9368764

[B5] Apelqvist A. Li H. Sommer L. Beatus P. Anderson D. J. Honjo T. (1999). Notch signalling controls pancreatic cell differentiation. *Nature* 400 877–881. 10.1038/23716 10476967

[B6] Bellusci S. Grindley J. Emoto H. Itoh N. Hogan B. L. (1997). Fibroblast growth factor 10 (FGF10) and branching morphogenesis in the embryonic mouse lung. *Development* 124 4867–4878. 9428423 10.1242/dev.124.23.4867

[B7] Benitez C. M. Goodyer W. R. Kim S. K. (2012). Deconstructing pancreas developmental biology. *Cold Spring Harb. Perspect. Biol.* 4:a012401. 10.1101/cshperspect.a012401 22587935 PMC3367550

[B8] Bhushan A. Itoh N. Kato S. Thiery J. P. Czernichow P. Bellusci S. (2001). Fgf10 is essential for maintaining the proliferative capacity of epithelial progenitor cells during early pancreatic organogenesis. *Development* 128 5109–5117. 11748146 10.1242/dev.128.24.5109

[B9] Burlison J. S. Long Q. Fujitani Y. Wright C. V. Magnuson M. A. (2008). Pdx-1 and Ptf1a concurrently determine fate specification of pancreatic multipotent progenitor cells. *Dev. Biol.* 316 74–86. 10.1016/j.ydbio.2008.01.011 18294628 PMC2425677

[B10] Celli G. LaRochelle W. J. Mackem S. Sharp R. Merlino G. (1998). Soluble dominant-negative receptor uncovers essential roles for fibroblast growth factors in multi-organ induction and patterning. *EMBO J.* 17 1642–1655. 10.1093/emboj/17.6.1642 9501086 PMC1170512

[B11] Chen N. M. Singh G. Koenig A. Liou G. Y. Storz P. Zhang J. S. (2015). NFATc1 Links EGFR Signaling to Induction of Sox9 Transcription and Acinar-Ductal Transdifferentiation in the Pancreas. *Gastroenterology* 148 1024.e9–1034.e9. 10.1053/j.gastro.2015.01.033 25623042 PMC4409493

[B12] Crisera C. A. Maldonado T. S. Kadison A. S. Li M. Alkasab S. L. Longaker M. T. (2000). Transforming growth factor-beta 1 in the developing mouse pancreas: a potential regulator of exocrine differentiation. *Differentiation* 65 255–259. 10.1046/j.1432-0436.2000.6550255.x 10929204

[B13] Desai T. J. Malpel S. Flentke G. R. Smith S. M. Cardoso W. V. (2004). Retinoic acid selectively regulates Fgf10 expression and maintains cell identity in the prospective lung field of the developing foregut. *Dev. Biol.* 273 402–415. 10.1016/j.ydbio.2004.04.039 15328022

[B14] Dong P. D. Munson C. A. Norton W. Crosnier C. Pan X. Gong Z. (2007). Fgf10 regulates hepatopancreatic ductal system patterning and differentiation. *Nat. Genet.* 39 397–402. 10.1038/ng1961 17259985

[B15] Edlund H. (1999). Pancreas: how to get there from the gut? *Curr. Opin. Cell Biol.* 11 663–668. 10.1016/S0955-0674(99)00033-2 10600706

[B16] Elghazi L. Cras-Meneur C. Czernichow P. Scharfmann R. (2002). Role for FGFR2IIIb-mediated signals in controlling pancreatic endocrine progenitor cell proliferation. *Proc. Natl. Acad. Sci. U.S.A.* 99 3884–3889. 10.1073/pnas.062321799 11891329 PMC122618

[B17] Francavilla C. Rigbolt K. T. Emdal K. B. Carraro G. Vernet E. Bekker-Jensen D. B. (2013). Functional proteomics defines the molecular switch underlying FGF receptor trafficking and cellular outputs. *Mol. Cell.* 51 707–722. 10.1016/j.molcel.2013.08.002 24011590

[B18] Friess H. Yamanaka Y. Buchler M. Berger H. G. Kobrin M. S. Baldwin R. L. (1993). Enhanced expression of the type II transforming growth factor beta receptor in human pancreatic cancer cells without alteration of type III receptor expression. *Cancer Res.* 53 2704–2707. 8389240

[B19] Fujikura J. Hosoda K. Iwakura H. Tomita T. Noguchi M. Masuzaki H. (2006). Notch/Rbp-j signaling prevents premature endocrine and ductal cell differentiation in the pancreas. *Cell Metab.* 3 59–65. 10.1016/j.cmet.2005.12.005 16399505

[B20] Fukuda A. Kawaguchi Y. Furuyama K. Kodama S. Horiguchi M. Kuhara T. (2008). Reduction of Ptf1a gene dosage causes pancreatic hypoplasia and diabetes in mice. *Diabetes Metab. Res. Rev.* 57 2421–2431. 10.2337/db07-1558 18591390 PMC2518493

[B21] Georgia S. Soliz R. Li M. Zhang P. Bhushan A. (2006). p57 and Hes1 coordinate cell cycle exit with self-renewal of pancreatic progenitors. *Dev. Biol.* 298 22–31. 10.1016/j.ydbio.2006.05.036 16899237

[B22] Givol D. Yayon A. (1992). Complexity of FGF receptors: genetic basis for structural diversity and functional specificity. *FASEB J.* 6 3362–3369. 10.1096/fasebj.6.15.1464370 1464370

[B23] Goetz R. Dover K. Laezza F. Shtraizent N. Huang X. Tchetchik D. (2009). Crystal structure of a fibroblast growth factor homologous factor (FHF) defines a conserved surface on FHFs for binding and modulation of voltage-gated sodium channels. *J. Biol. Chem.* 284 17883–17896. 10.1074/jbc.M109.001842 19406745 PMC2719427

[B24] Goetz R. Mohammadi M. (2013). Exploring mechanisms of FGF signalling through the lens of structural biology. *Nat. Rev. Mol. Cell Biol.* 14 166–180. 10.1038/nrm3528 23403721 PMC3695728

[B25] Gouzi M. Kim Y. H. Katsumoto K. Johansson K. Grapin-Botton A. (2011). Neurogenin3 initiates stepwise delamination of differentiating endocrine cells during pancreas development. *Dev. Dyn.* 240 589–604. 10.1002/dvdy.22544 21287656

[B26] Greggio C. De Franceschi F. Figueiredo-Larsen M. Gobaa S. Ranga A. Semb H. (2013). Artificial three-dimensional niches deconstruct pancreas development in vitro. *Development* 140 4452–4462. 10.1242/dev.096628 24130330 PMC4007719

[B27] Grimont A. Pinho A. V. Cowley M. J. Augereau C. Mawson A. Giry-Laterriere M. (2015). SOX9 regulates ERBB signalling in pancreatic cancer development. *Gut* 64 1790–1799. 10.1136/gutjnl-2014-307075 25336113

[B28] Guo L. Degenstein L. Fuchs E. (1996). Keratinocyte growth factor is required for hair development but not for wound healing. *Genes Dev.* 10 165–175. 10.1101/gad.10.2.165 8566750

[B29] Hart A. Papadopoulou S. Edlund H. (2003). Fgf10 maintains notch activation, stimulates proliferation, and blocks differentiation of pancreatic epithelial cells. *Dev. Dyn.* 228 185–193. 10.1002/dvdy.10368 14517990

[B30] Heller R. S. Dichmann D. S. Jensen J. Miller C. Wong G. Madsen O. D. (2002). Expression patterns of Wnts, Frizzleds, sFRPs, and misexpression in transgenic mice suggesting a role for Wnts in pancreas and foregut pattern formation. *Dev. Dyn.* 225 260–270. 10.1002/dvdy.10157 12412008

[B31] Hessmann E. Zhang J. S. Chen N. M. Hasselluhn M. Liou G. Y. Storz P. (2016). NFATc4 regulates Sox9 gene expression in acinar cell plasticity and pancreatic cancer initiation. *Stem Cells Int.* 2016:5272498. 10.1155/2016/5272498 26697077 PMC4677249

[B32] Holzmann K. Grunt T. Heinzle C. Sampl S. Steinhoff H. Reichmann N. (2012). Alternative splicing of fibroblast growth factor receptor IgIII loops in cancer. *J. Nucleic Acids* 2012:950508. 10.1155/2012/950508 22203889 PMC3238399

[B33] Hrabe de Angelis M. McIntyre J. II Gossler A. (1997). Maintenance of somite borders in mice requires the Delta homologue DII1. *Nature* 386 717–721. 10.1038/386717a0 9109488

[B34] Itoh N. Ohta H. (2014). Fgf10: a paracrine-signaling molecule in development, disease, and regenerative medicine. *Curr. Mol. Med.* 14 504–509. 10.2174/1566524014666140414204829 24730525

[B35] Itoh N. Ornitz D. M. (2011). Fibroblast growth factors: from molecular evolution to roles in development, metabolism and disease. *J. Biochem.* 149 121–130. 10.1093/jb/mvq121 20940169 PMC3106964

[B36] Jensen J. Pedersen E. E. Galante P. Hald J. Heller R. S. Ishibashi M. (2000). Control of endodermal endocrine development by Hes-1. *Nat. Genet.* 24 36–44. 10.1038/71657 10615124

[B37] Jonckheere N. Mayes E. Shih H. P. Li B. Lioubinski O. Dai X. (2008). Analysis of mPygo2 mutant mice suggests a requirement for mesenchymal Wnt signaling in pancreatic growth and differentiation. *Dev. Biol.* 318 224–235. 10.1016/j.ydbio.2008.03.014 18452912 PMC2478757

[B38] Jorgensen M. C. Ahnfelt-Ronne J. Hald J. Madsen O. D. Serup P. Hecksher-Sorensen J. (2007). An illustrated review of early pancreas development in the mouse. *Endocr. Rev.* 28 685–705. 10.1210/er.2007-0016 17881611

[B39] Kalluri R. Zeisberg M. (2006). Fibroblasts in cancer. *Nat. Rev. Cancer* 6 392–401. 10.1038/nrc1877 16572188

[B40] Kato S. Sekine K. (1999). FGF-FGFR signaling in vertebrate organogenesis. *Cell Mol. Biol.* 45 631–638. 10512194

[B41] Kawaguchi Y. Cooper B. Gannon M. Ray M. MacDonald R. J. Wright C. V. (2002). The role of the transcriptional regulator Ptf1a in converting intestinal to pancreatic progenitors. *Nat. Genet.* 32 128–134. 10.1038/ng959 12185368

[B42] Kawahira H. Scheel D. W. Smith S. B. German M. S. Hebrok M. (2005). Hedgehog signaling regulates expansion of pancreatic epithelial cells. *Dev. Biol.* 280 111–121. 10.1016/j.ydbio.2005.01.008 15766752

[B43] Kobberup S. Nyeng P. Juhl K. Hutton J. Jensen J. (2007). ETS-family genes in pancreatic development. *Dev. Dyn.* 236 3100–3110. 10.1002/dvdy.21292 17907201

[B44] Kobberup S. Schmerr M. Dang M. L. Nyeng P. Jensen J. N. MacDonald R. J. (2010). Conditional control of the differentiation competence of pancreatic endocrine and ductal cells by Fgf10. *Mech. Dev.* 127 220–234. 10.1016/j.mod.2009.11.005 19969077 PMC2849919

[B45] Kopp J. L. von Figura G. Mayes E. Liu F. F. Dubois C. L. Morris J. P. (2012). Identification of Sox9-dependent acinar-to-ductal reprogramming as the principal mechanism for initiation of pancreatic ductal adenocarcinoma. *Cancer Cell* 22 737–750. 10.1016/j.ccr.2012.10.025 23201164 PMC3568632

[B46] Landsman L. Nijagal A. Whitchurch T. J. Vanderlaan R. L. Zimmer W. E. Mackenzie T. C. (2011). Pancreatic mesenchyme regulates epithelial organogenesis throughout development. *PLoS Biol.* 9:e1001143. 10.1371/journal.pbio.1001143 21909240 PMC3167782

[B47] Lindahl U. Kusche-Gullberg M. Kjellen L. (1998). Regulated diversity of heparan sulfate. *J. Biol. Chem.* 273 24979–24982. 10.1074/jbc.273.39.24979 9737951

[B48] Madsen O. D. Serup P. (2006). Towards cell therapy for diabetes. *Nat. Biotechnol.* 24 1481–1483. 10.1038/nbt1206-1481 17160042

[B49] Makarenkova H. P. Hoffman M. P. Beenken A. Eliseenkova A. V. Meech R. Tsau C. (2009). Differential interactions of FGFs with heparan sulfate control gradient formation and branching morphogenesis. *Sci. Signal.* 2:ra55. 10.1126/scisignal.2000304 19755711 PMC2884999

[B50] Martin M. Gallego-Llamas J. Ribes V. Kedinger M. Niederreither K. Chambon P. (2005). Dorsal pancreas agenesis in retinoic acid-deficient Raldh2 mutant mice. *Dev. Biol.* 284 399–411. 10.1016/j.ydbio.2005.05.035 16026781

[B51] Mfopou J. K. Chen B. Mateizel I. Sermon K. Bouwens L. (2010). Noggin, retinoids, and fibroblast growth factor regulate hepatic or pancreatic fate of human embryonic stem cells. *Gastroenterology* 138 2233.e14–2245.e14. 10.1053/j.gastro.2010.02.056 20206178

[B52] Min H. Danilenko D. M. Scully S. A. Bolon B. Ring B. D. Tarpley J. E. (1998). Fgf-10 is required for both limb and lung development and exhibits striking functional similarity to Drosophila branchless. *Genes Dev.* 12 3156–3161. 10.1101/gad.12.20.3156 9784490 PMC317210

[B53] Miralles F. Czernichow P. Ozaki K. Itoh N. Scharfmann R. (1999). Signaling through fibroblast growth factor receptor 2b plays a key role in the development of the exocrine pancreas. *Proc. Natl. Acad. Sci. U.S.A.* 96 6267–6272. 10.1073/pnas.96.11.6267 10339576 PMC26870

[B54] Miralles F. Lamotte L. Couton D. Joshi R. L. (2006). Interplay between FGF10 and Notch signalling is required for the self-renewal of pancreatic progenitors. *Int. J. Dev. Biol.* 50 17–26. 10.1387/ijdb.052080fm 16323074

[B55] Moustakas A. Heldin C. H. (2007). Signaling networks guiding epithelial-mesenchymal transitions during embryogenesis and cancer progression. *Cancer Sci.* 98 1512–1520. 10.1111/j.1349-7006.2007.00550.x 17645776 PMC11158989

[B56] Murtaugh L. C. Stanger B. Z. Kwan K. M. Melton D. A. (2003). Notch signaling controls multiple steps of pancreatic differentiation. *Proc. Natl. Acad. Sci. U.S.A.* 100 14920–14925. 10.1073/pnas.2436557100 14657333 PMC299853

[B57] Nomura S. Yoshitomi H. Takano S. Shida T. Kobayashi S. Ohtsuka M. (2008). FGF10/FGFR2 signal induces cell migration and invasion in pancreatic cancer. *Br. J. Cancer* 99 305–313. 10.1038/sj.bjc.6604473 18594526 PMC2480967

[B58] Norgaard G. A. Jensen J. N. Jensen J. (2003). FGF10 signaling maintains the pancreatic progenitor cell state revealing a novel role of Notch in organ development. *Dev. Biol.* 264 323–338. 10.1016/j.ydbio.2003.08.013 14651921

[B59] Offield M. F. Jetton T. L. Labosky P. A. Ray M. Stein R. W. Magnuson M. A. (1996). PDX-1 is required for pancreatic outgrowth and differentiation of the rostral duodenum. *Development* 122 983–995. 8631275 10.1242/dev.122.3.983

[B60] Ohuchi H. Hori Y. Yamasaki M. Harada H. Sekine K. Kato S. (2000). FGF10 acts as a major ligand for FGF receptor 2 IIIb in mouse multi-organ development. *Biochem. Biophys. Res. Commun.* 277 643–649. 10.1006/bbrc.2000.3721 11062007

[B61] Ornitz D. M. Itoh N. (2015). The Fibroblast Growth Factor signaling pathway. *Wiley Interdiscip. Rev. Dev. Biol.* 4 215–266. 10.1002/wdev.176 25772309 PMC4393358

[B62] Orr-Urtreger A. Bedford M. T. Burakova T. Arman E. Zimmer Y. Yayon A. (1993). Developmental localization of the splicing alternatives of fibroblast growth factor receptor-2 (FGFR2). *Dev. Biol.* 158 475–486. 10.1006/dbio.1993.1205 8393815

[B63] O’Sullivan H. Kelleher F. C. Lavelle M. McGovern B. Murphy J. Swan N. (2017). Therapeutic potential for FGFR inhibitors in SOX9-FGFR2 coexpressing pancreatic cancer. *Pancreas* 46 e67–e69. 10.1097/MPA.0000000000000870 28796141

[B64] Pan F. C. Wright C. (2011). Pancreas organogenesis: from bud to plexus to gland. *Dev. Dyn.* 240 530–565. 10.1002/dvdy.22584 21337462

[B65] Papadopoulou S. Edlund H. (2005). Attenuated Wnt signaling perturbs pancreatic growth but not pancreatic function. *Diabetes Metab. Res. Rev.* 54 2844–2851. 16186384 10.2337/diabetes.54.10.2844

[B66] Petri A. Ahnfelt-Ronne J. Frederiksen K. S. Edwards D. G. Madsen D. Serup P. (2006). The effect of neurogenin3 deficiency on pancreatic gene expression in embryonic mice. *J. Mol. Endocrinol.* 37 301–316. 10.1677/jme.1.02096 17032746

[B67] Pictet R. L. Clark W. R. Williams R. H. Rutter W. J. (1972). An ultrastructural analysis of the developing embryonic pancreas. *Dev. Biol.* 29 436–467. 10.1016/0012-1606(72)90083-8 4570759

[B68] Pulkkinen M. A. Spencer-Dene B. Dickson C. Otonkoski T. (2003). The IIIb isoform of fibroblast growth factor receptor 2 is required for proper growth and branching of pancreatic ductal epithelium but not for differentiation of exocrine or endocrine cells. *Mech. Dev.* 120 167–175. 10.1016/S0925-4773(02)00440-9 12559489

[B69] Seiki M. (2003). Membrane-type 1 matrix metalloproteinase: a key enzyme for tumor invasion. *Cancer Lett.* 194 1–11. 10.1016/S0304-3835(02)00699-7 12706853

[B70] Sekine K. Ohuchi H. Fujiwara M. Yamasaki M. Yoshizawa T. Sato T. (1999). Fgf10 is essential for limb and lung formation. *Nat. Genet.* 21 138–141. 10.1038/5096 9916808

[B71] Seymour P. A. (2014). Sox9: a master regulator of the pancreatic program. *Rev. Diabetes Stud.* 11 51–83. 10.1900/RDS.2014.11.51 25148367 PMC4295800

[B72] Seymour P. A. Shih H. P. Patel N. A. Freude K. K. Xie R. Lim C. J. (2012). A Sox9/Fgf feed-forward loop maintains pancreatic organ identity. *Development* 139 3363–3372. 10.1242/dev.078733 22874919 PMC3424044

[B73] Shih H. P. Seymour P. A. Patel N. A. Xie R. Wang A. Liu P. P. (2015). A gene regulatory network cooperatively controlled by Pdx1 and Sox9 governs lineage allocation of foregut progenitor cells. *Cell Rep.* 13 326–336. 10.1016/j.celrep.2015.08.082 26440894 PMC4607666

[B74] Shih H. P. Wang A. Sander M. (2013). Pancreas organogenesis: from lineage determination to morphogenesis. *Annu. Rev. Cell Dev. Biol.* 29 81–105. 10.1146/annurev-cellbio-101512-122405 23909279

[B75] Stafford D. White R. J. Kinkel M. D. Linville A. Schilling T. F. Prince V. E. (2006). Retinoids signal directly to zebrafish endoderm to specify insulin-expressing beta-cells. *Development* 133 949–956. 10.1242/dev.02263 16452093

[B76] Stoffers D. A. Zinkin N. T. Stanojevic V. Clarke W. L. Habener J. F. (1997). Pancreatic agenesis attributable to a single nucleotide deletion in the human IPF1 gene coding sequence. *Nat. Genet.* 15 106–110. 10.1038/ng0197-106 8988180

[B77] Tefft D. Lee M. Smith S. Crowe D. L. Bellusci S. Warburton D. (2002). mSprouty2 inhibits FGF10-activated MAP kinase by differentially binding to upstream target proteins. *Am. J. Physiol. Lung Cell Mol. Physiol.* 283 L700–L706. 10.1152/ajplung.00372.2001 12225946

[B78] ten Berge D. Brugmann S. A. Helms J. A. Nusse R. (2008). Wnt and FGF signals interact to coordinate growth with cell fate specification during limb development. *Development* 135 3247–3257. 10.1242/dev.023176 18776145 PMC2756806

[B79] Wiebe P. O. Kormish J. D. Roper V. T. Fujitani Y. Alston N. I. Zaret K. S. (2007). Ptf1a binds to and activates area III, a highly conserved region of the Pdx1 promoter that mediates early pancreas-wide Pdx1 expression. *Mol. Cell. Biol.* 27 4093–4104. 10.1128/MCB.01978-06 17403901 PMC1900007

[B80] Ye F. Duvillie B. Scharfmann R. (2005). Fibroblast growth factors 7 and 10 are expressed in the human embryonic pancreatic mesenchyme and promote the proliferation of embryonic pancreatic epithelial cells. *Diabetologia* 48 277–281. 10.1007/s00125-004-1638-6 15690149

[B81] Ying H. Dey P. Yao W. Kimmelman A. C. Draetta G. F. Maitra A. (2016). Genetics and biology of pancreatic ductal adenocarcinoma. *Genes Dev.* 30 355–385. 10.1101/gad.275776.115 26883357 PMC4762423

[B82] Zhang X. Ibrahimi O. A. Olsen S. K. Umemori H. Mohammadi M. Ornitz D. M. (2006). Receptor specificity of the fibroblast growth factor family. The complete mammalian FGF family. *J. Biol. Chem.* 281 15694–15700. 10.1074/jbc.M601252200 16597617 PMC2080618

[B83] Zhou Q. Law A. C. Rajagopal J. Anderson W. J. Gray P. A. Melton D. A. (2007). A multipotent progenitor domain guides pancreatic organogenesis. *Dev. Cell* 13 103–114. 10.1016/j.devcel.2007.06.001 17609113

[B84] Zhou H. Qin Y. Ji S. Ling J. Fu J. Zhuang Z. (2018). SOX9 activity is induced by oncogenic Kras to affect MDC1 and MCMs expression in pancreatic cancer. *Oncogene* 37 912–923. 10.1038/onc.2017.393 29059173 PMC6545484

[B85] Zinkle A. Mohammadi M. (2018). A threshold model for receptor tyrosine kinase signaling specificity and cell fate determination. *F1000Res* 7:F1000FacultyRev–872. 29983915 10.12688/f1000research.14143.1 PMC6013765

